# Performance of the BACES model in a Dutch cohort with nontuberculous mycobacterial pulmonary disease

**DOI:** 10.1016/j.jctube.2026.100628

**Published:** 2026-06-27

**Authors:** Arthur Lemson, Esmee van de Pol, Jakko van Ingen, Wouter Hoefsloot

**Affiliations:** a Department of Pulmonary Diseases, Research Institute for Medical Innovation, Radboud University Medical Center, Radboudumc Community for Infectious Diseases, Nijmegen, the Netherlands; b Department of Medical Microbiology, Radboudumc Community for Infectious Diseases, Radboud University Medical Center, Nijmegen, the Netherlands

**Keywords:** All-cause mortality, BACES Model, External Validation, *Mycobacterium avium* complex, *Mycobacterium abscessus*

## Abstract

**Objectives:**

The BACES model, developed in South Korea, predicts all-cause mortality in patients with nontuberculous mycobacterial pulmonary disease (NTM-PD) using BMI, age, cavitary disease, erythrocyte sedimentation rate, and male sex. The total score categorizes patients into low, intermediate, or high mortality risk groups. We evaluated the performance of BACES in a Dutch cohort of patients with NTM-PD.

**Methods:**

This retrospective analysis included adults with NTM-PD between January 2008 and August 2019. Time to all-cause mortality was calculated from diagnosis. Performance of the original and modified BACES models were assessed using Kaplan-Meier curves, Harrell's C-statistic, and calibration curves. Cox regression identified independent mortality predictors.

**Results:**

We enrolled 183 patients (median age 63 years, 56% female), of whom 86.3% received antimycobacterial therapy. The median (IQR) follow-up time was 6.3 years (3.7–9.0) with 54.1% deaths. BACES risk scores categorized 118 patients: 23.7% low risk, 63.3% intermediate risk, and 12.7% high risk. Among risk groups, Kaplan-Meier curves demonstrated clear stratification (log-rank <0.001). Harrell's C-statistic and calibration curves were evaluated in 86 patients due to missing data. Harrell's C-statistic was 0.67 (95%CI, 0.57–0.76), indicating poor discrimination, but the calibration curve showed good agreement between predicted and observed mortality. After modifying the model by adjusting the ESR cut-off and replacing sex with COPD history, the C-statistic improved to 0.76 (95% CI 0.69–0.84).

**Conclusions:**

The BACES model proves useful for group-level risk stratification (e.g., low, intermediate high), but lacks precision in predicting individual prognosis in Dutch patients with NTM-PD. Modifications to the model enhanced its discriminatory performance.

## 1 Introduction

Nontuberculous mycobacterial pulmonary disease (NTM-PD) is an emerging health problem [1]. Its clinical course shows important interindividual variation, requiring personalized treatment strategies [2]. The appropriate treatment strategy is determined by a comprehensive assessment, considering the causative NTM species, drug susceptibility, prognosis, and patient preferences.

Estimating the prognosis in clinical practice is challenging. Some patients remain clinically stable without antimycobacterial treatment and may even clear the NTM spontaneously, while others deteriorate and require treatment initiation at a worse condition [3], [4], [5], [6]. To inform on the prognosis, a prediction model for time to all-cause mortality was developed and validated in a large South Korean NTM-PD cohort [7]. The so-called BACES model assigns one point for the following covariates: BMI < 18.5 kg/m^2^, age ≥ 65 years, cavitary disease, erythrocyte sedimentation rate (ESR) ≥ 15 mm/h in males and ≥ 20 mm/h in females, and male sex, resulting in a total score that ranges from 0 to 5. Furthermore, the total score can be categorized a low risk (score 0–1), intermediate risk (score 2–3), and high risk (score 4–5) group.

External validation of prediction models is essential for implementation into clinical practice but is often challenged by inherent differences between populations and healthcare systems. The performance of the BACES model was recently evaluated in a retrospective NTM-PD cohort from Canada. The model demonstrated moderate to good discrimination performance, but consistently underpredicted all-cause mortality, indicating suboptimal calibration [8]. An exploratory analysis revealed that adjusting the BMI threshold to 19.5 kg/m^2^ slightly improved the calibration, though no further optimization of the model was performed.

We aimed to a) study the performance of the original BACES model in predicting time to all-cause mortality for an NTM-PD cohort from the Netherlands and b) identify other predictors to potentially optimize the model.

## 2 Methods

### 2.1 Study design and participants

We conducted a single-center, retrospective chart review at Radboudumc in the Netherlands, the national reference clinic for NTM disease. Adults diagnosed with *M. avium* complex (MAC) or *M. abscessus* (MAB) pulmonary disease that attended our clinic between January 1, 2008, and August 1, 2019, were considered eligible. Patients with a concurrent diagnosis of cystic fibrosis or human immunodeficiency virus infection were excluded, as well as those who declined the possibility to be contacted for participation in future research upon hospital registration. The time window was chosen to ensure a minimal 5-year follow-up time from diagnosis.

Written informed consent was not required due to the retrospective design, thus verbal informed consent was obtained from those alive and with up-to-date contact details, in accordance with local standard of practice. Consent was assumed in deceased patients or those without up-to-date contact details. The study was exempt from approval by the institutional review board (METC Oost-Nederland; file number: 2024–17038).

The primary outcome was all-cause mortality. Date of death was retrieved from the hospital's electronic health record or obtained from the general practitioner. Participants were followed until August 1, 2024, and censored at their last known contact with Radboudumc, the referring physician, or a general practitioner. Time to all-cause mortality was calculated from the date of NTM-PD diagnosis, defined as the date of the first positive NTM culture in those fulfilling all diagnostic criteria [9]. In case the date of the first positive NTM culture was unknown, the date of the first reported NTM-PD diagnosis by the referring pulmonologist or Radboudumc employed pulmonologist was used.

Since Radboudumc is a tertiary reference clinic for NTM disease, some of the patients received NTM-PD treatment prior to referral.

### 2.2 Data extraction

Data were retrieved from the hospital's electronic health record. The BACES components at or closest to the time of NTM-PD diagnosis were recorded. To account for our role as reference clinic, we allowed BACES values (i.e., BMI, presence of a cavitary lesion, and ESR) from one year prior to diagnosis up to one year after, and no longer than 3 months after antimycobacterial therapy was initiated. Radiological disease manifestation was classified by the authors (AL and WH) and discussed with an independent radiologist in case of uncertainty. Other collected variables included smoking history, comorbidities, biochemistry, MAC or MAB subspecies, acid-fast bacilli smear status, pulmonary function testing, NTM-PD treatment, and treatment outcomes. We used treatment outcome definitions as stated in the NTM-NET consensus statement [10], with the exception of culture conversion, which was not limited to 6 months of treatment and defined as ≥2 consecutive negative sputum cultures or one negative culture from bronchoscopy.

### 2.3 Statistical analysis

Data analyses were carried out in SPSS version 29 and RStudio version 1.1.463. Categorial data were reported as counts and proportions, continuous variables were expressed median (interquartile range, 25th–75th percentile).

#### 2.3.1 BACES score

A BACES score was determined for participants with all five components available. Participants with a complete BACES score entered the formal validation analysis. Participants with one missing BACES component summing up to an incomplete score of 0, 2, or 4 were included in the BACES risk group analysis, as including the fifth missing component would not have resulted in classification into a different risk group. Participants without a complete BACES score were included in the analysis of secondary endpoints.

#### 2.3.2 Discriminatory performance and calibration

The performance of a survival prediction model in an external cohort is determined by assessing the discriminatory and calibration performance [11]. Discriminatory performance refers to the model's ability to distinguish between individual prognoses. Discrimination was informally evaluated by visual assessment of Kaplan-Meier survival curves and comparing risk groups with the log-rank test. Furthermore, hazard ratios for all-cause mortality were compared between risk groups using Cox regression. Next, regression coefficients from the original BACES model description [7] were used to determine the model's prognostic index through Cox proportional hazard regression, and subsequently, compute the Harrell's concordance (C-) index, which reflects the proportion of pairs for which the model correctly predicts the order of events. Calibration performance reflects the agreement between the model's predicted risk and the observed risk. Calibration was visualized by comparing the predicted all-cause mortality rate with the observed all-cause mortality rate for each BACES risk group from the present cohort.

#### 2.3.3 Model optimization

After analyzing the performance of the original BACES model, we modified the ESR cut-offs to align with reference values in The Netherlands by multiplying the local upper limit of normal, 20 mm/h in males and 30 mm/h in females, by 1.5 (i.e., modified ESR elevated if ≥30 mm/h in males and ≥ 45 mm/h in females). Next, univariate and multivariate Cox regression were performed to 1) determine the predictive ability of individual BACES components for all-cause mortality and 2) identify additional predictors of all-cause mortality that could optimize performance of the BACES model using stepwise (forward) selection and a 5% significance level. Regression coefficients from the multivariate Cox model were used to compute the Harrell's C-statistic and calibration curve of the modified BACES model. The Cox proportional hazard assumption was checked for all covariates included in (a modified version of) the BACES model by plotting the Schoenfeld residuals.

## 3 Results

### 3.1 Population characteristics and outcomes

We included 183 patients in the study, with a median age of 63 years (IQR, 55–70) and 103 (56%) were female. MAC was the causative agent in 161 cases (88%), of which 93 (57.8%) were identified as *M. avium*. MAB was isolated in 22 (12%) participants, with 3 (13.6%) identified as subspecies *massiliense*. All population characteristics at the time of NTM-PD diagnosis are summarized in Table 1.

**Table 1:** Population characteristics at NTM-PD diagnosis.

Characteristics	Median (IQR) or proportion (%)			
	Overall (*N* = 183)	Low risk^⁎^ (*N* = 28)	Intermediate^⁎^ risk (*N* = 75)	High risk^⁎^ (*N* = 15)
Follow-up period until death or censoring, years	6.3 (3.7–9.0)	6.8 (4.5–10.9)	6.2 (3.7–8.2)	1.6 (0.7–4.3)
BACES components				
BMI, kg/m^2^	21.0 (18.4–23.4)	22.2 (20.2–23.7)	20.6 (18.2–22.9)	18.9 (17.4–22.2)
Age at diagnosis, years	63 (55–70)	57 (48.3–63)	66 (55–71)	70 (65–71)
Presence of cavity	91 (49.7%)	4 (14.3%)	43 (57.3%)	15 (100%)
Erythrocyte sedimentation rate, mm/h	28.0 (12–57)	10 (5.5–18.5)	32 (17.3–61.5)	45 (26–96)
Sex, male	80 (43.7%)	4 (14.3%)	30 (40%)	14 (93.3%)
Smoking history				
Current	64 (36.8%)	6 (22.2%)	28 (38.9%)	11 (73.3%)
Former	75 (43.1%)	13 (48.1%)	29 (40.3%)	4 (26.7%)
Never	35 (20.1%)	8 (29.6%)	15 (20.8%)	0
Comorbidity				
COPD	97 (53%)	11 (39.3%)	41 (54.7%)	11 (73.3%)
Cardiovascular disease	56 (30.6%)	10 (35.7%)	21 (28%)	8 (53.3%)
History of bronchiectasis prior to NTM-PD	40 (21.9%)	4 (14.3%)	18 (24%)	0
Former or current alcohol abuse	31 (16.9%)	0	12 (16%)	8 (53.3%)
Asthma	30 (16.4%)	6 (21.4%)	13 (17.3%)	1 (6.7%)
History of malignancy	26 (14.2%)	5 (17.9%)	15 (20%)	2 (13.3%)
Immunocompromised status	21 (11.5%)	2 (7.1%)	11 (14.7%)	0
Pulmonary aspergillosis	15 (8.2%)	2 (7.1%)	6 (8%)	0
History of tuberculosis infection	12 (6.6%)	2 (7.1%)	5 (6.7%)	1 (6.7%)
Diabetes mellitus	7 (3.8%)	0	3 (4%)	1 (6.7%)
Interstitial lung disease	5 (2.7%)	2 (7.1%)	3 (4%)	0
Active malignancy	4 (2.2%)	0	3 (4%)	1 (6.7%)
Liver disease	2 (1.1%)	0	0	1 (6.3%)
Alfa-1-antitrypsine deficiency	2 (1.1%)	0	1 (1.3%)	0
NTM species				
*M. avium* complex	161 (88%)	24 (85.7%)	66 (88%)	12 (80%)
*M. abscessus*	22 (12%)	4 (14.3%)	9 (12%)	3 (20%)
Acid-fast bacilli smear positive	72 (51.1%)	11 (47.8%)	32 (47.8%)	9 (57.1%)
Dominant disease manifestation				
Nodular-bronchiectatic	75 (41%)	21 (75%)	24 (32%)	0
Fibrocavitary	35 (19.1%)	1 (3.6%)	19 (25.3%)	6 (40%)
Nodular-bronchiectatic and (fibro)cavitary	57 (31.1%)	3 (10.7%)	24 (32%)	9 (60%)
Unspecified	16 (8.7%)	3 (10.7%)	8 (10.7%)	0

*Abbreviations: BMI = Body Mass Index; COPD = Chronic Obstructive Pulmonary Disease.*^⁎^BACES risk groups: low risk (score 0–1), intermediate risk (2–3), and high risk (score 4–5).

Data on 5-year mortality was available for 180 (98.4%) patients (Table 2). The median follow-up time until death or censoring was 6.3 years (IQR, 3.7–9.0). During the follow-up period, 99 (54.1%) participants died, with a median survival time of 4.0 years (IQR, 1.8–6.9) and 5-year all-cause mortality rate of 33.3%. Notably, antimycobacterial therapy was initiated in 158 (86.3%) participants with a median treatment duration of 14 months (IQR, 8.3–21.8). The microbiological cure rate among treated participants with follow-up data available was 71.6% (96/134) (Table 2).

**Table 2:** Treatment and outcomes in all participants.

Treatment and outcomes in all participants (N = 183)	Median (IQR) or proportion (%)
NTM-PD treatment	
Antimycobacterial treatment initiation	158 (86.3%)
Time to treatment initiation, months	2.5 (1–9)
Total treatment duration, months	14 (8.3–21.8)
Adjuvant lung surgery	21 (11.5%)
Treatment outcomes^⁎^	
Culture conversion^⁎⁎^	109 (85.2%)
Time to culture conversion, months	5.0 (2–15)
Spontaneous culture conversion	22 (73.3%)
Time to spontaneous culture conversion, months	7.8 (3–24.3)
Clinical improvement	102 (65%)
Microbiological cure	96 (71.6%)
Cure	78 (59.1%)
Recurrence After Treatment	27 (45.8%)
All-cause mortality	
Death during follow-up	99 (54.1%)
Death within 5 years after diagnosis	60 (33.3%)
Death within 10 years after diagnosis	88 (71.0%)
Death within 15 years after diagnosis	93 (87.7%)

Abbreviations: NTM-PD = nontuberculous mycobacterial pulmonary disease. ^⁎^As defined in the NTM-NET consensus statement [10]. ^⁎⁎^Defined as ≥2 consecutive negative sputum cultures or one negative culture from bronchoscopy.

**Fig. 1:**
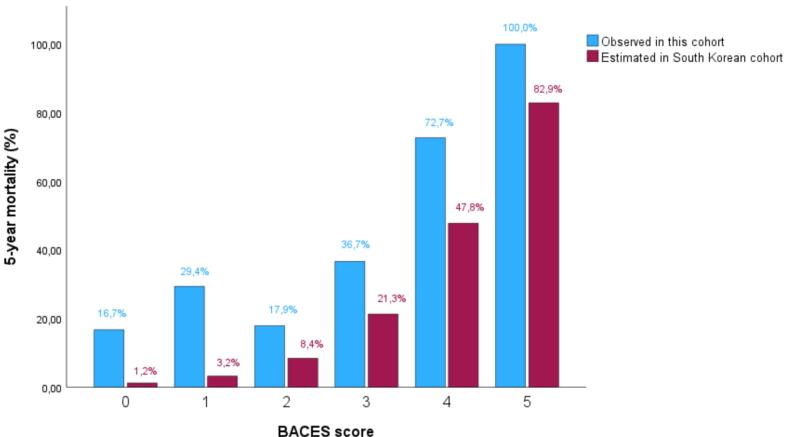
Observed 5-year mortality rate by BACES score in the Dutch cohort (blue bars) vs. estimated 5-year mortality rate by BACES score in the South Korean cohort (red bars). (For interpretation of the references to colour in this figure legend, the reader is referred to the web version of this article.)

A complete BACES score was calculated for 98 patients (53.6%) (see Table 1 & fig. S1). Among these, 25 participants (25.6%) were classified as low risk, 59 (60.2%) as intermediate risk, and 14 (14.3%) as high risk. Including twenty patients with incomplete BACES scores (i.e., scores of 0, 2, or 4) increased the group sizes to 28 (23.7%), 75 (63.3%), and 15 (12.7%), respectively. The observed 5-year mortality rates across BACES scores within our cohort were plotted against the estimated 5-year mortality rates from South Korea [7] (see Fig. 1), revealing underprediction throughout all BACES scores, particularly in the low risk group.

### 3.2 Discriminatory performance and calibration

**Fig. 2:**
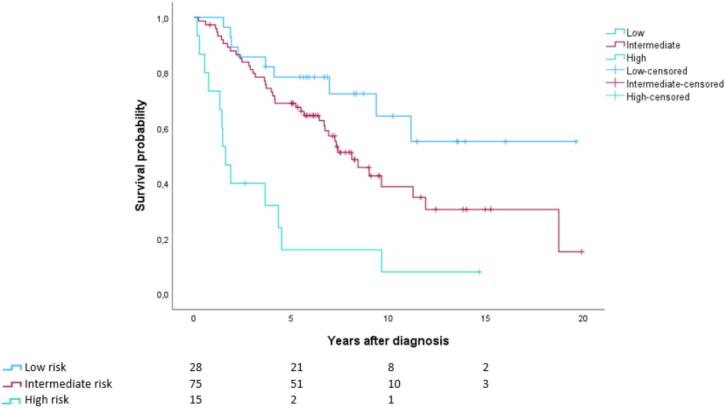
Kaplan-Meier survival curves stratified by BACES risk groups.

Among the 118 patients with a known BACES risk score, 62 died during follow-up. Kaplan-Meier survival curves were well-separated between risk groups (log-rank <0.001), except for the low and intermediate risk group during the first 3 years after diagnosis (Fig. 2). Hazard ratios for time to all-cause mortality between risk groups were compared using Cox regression: 1.92 (95% CI, 0.95–4.05; *p* = 0.068) for intermediate vs low risk, 3.23 (95% CI, 1.71–6.12; *p* < 0.001) for high vs. intermediate risk, and 6.36 (95% CI, 2.70–14.97; p < 0.001) for high vs. low risk groups.

**Fig. 3:**
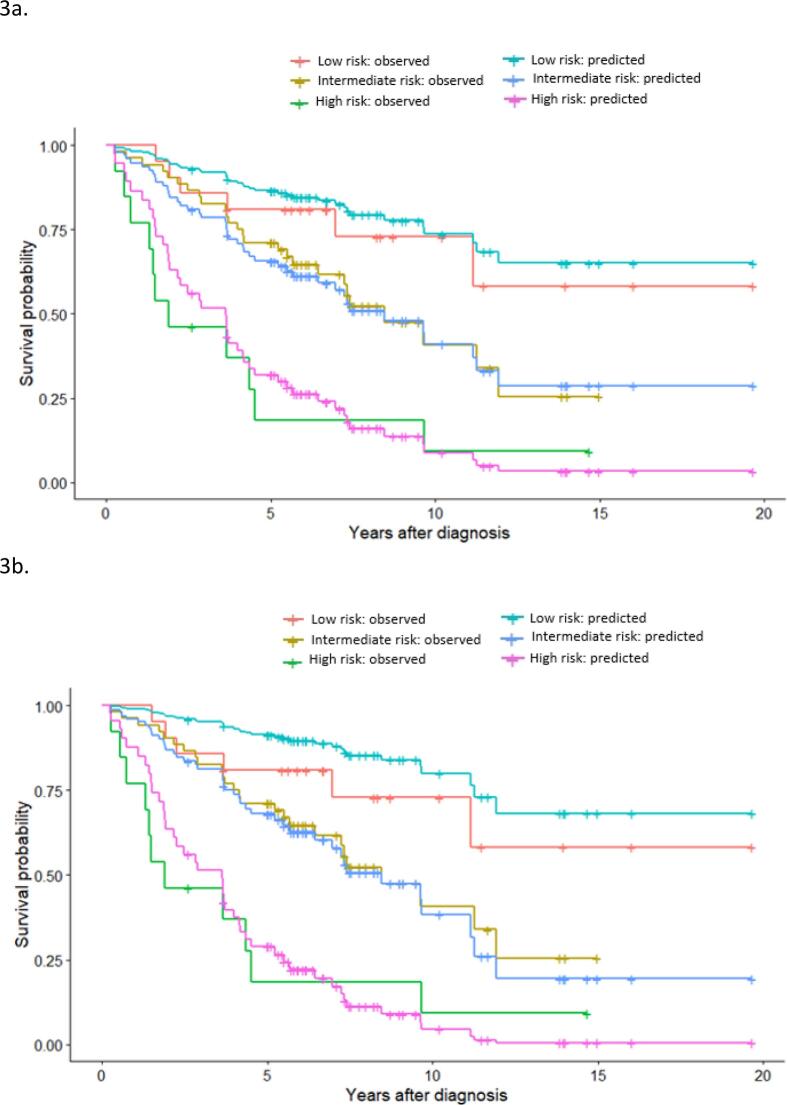
Calibration curves comparing predicted to observed all-cause mortality for the original (a) and modified (b) BACES model.

The Harrell C-statistic was calculated for 86 patients (43 deaths) with a complete total BACES score and known acid-fast bacilli smear status, and found 0.67 (95% CI, 0.57–0.76), indicating poor discriminatory performance. Conversely, calibration curves of the BACES risk groups (*N* = 86) demonstrated good agreement between predicted and observed all-cause mortality (Fig. 3a). A comparison between patients included (N = 86) and those excluded (*N* = 96) from the validation analyses revealed no clinically significant differences in baseline characteristics or mortality rates.

### 3.3 Model optimization

Multivariate Cox regression identified three components of the original BACES model (age, BMI, and cavitation) as significant predictors of time to all-cause mortality (table S2). After modifying the ESR cut-off to align with the Dutch standard, its predictive ability improved sufficiently to be included in the optimized model (table S3). Additionally, Cox regression demonstrated that a history of COPD and cardiovascular disease were significant independent predictors of time to all-cause mortality, although cardiovascular disease was excluded from the final multivariate model due to violation of the proportional hazards' assumption (table S3).

The discriminatory performance of the modified BACES model, including age ≥ 65 years, BMI < 18.5 kg/m2, cavitation, ESR ≥ 30 in males and ≥ 45 in females, and COPD history, was re-assessed with Harrell's C-statistic, yielding an improved value of 0.76 (95%CI 0.69–0.84), indicating moderate discrimination. The modified model demonstrated a minor improvement in calibration for the intermediate, but not the low and high risk groups (Fig. 3b).

The predictive power of other baseline characteristics such as biochemistry (e.g., hemoglobin, leukocytes) and pulmonary function testing (e.g., FEV1) were explored using univariate cox regression, but not included in the multivariate model due to missing information (table S4).

## 4 Discussion

This study is the second to evaluate the performance of the BACES survival prediction model in an external NTM-PD cohort. While the model demonstrated a significant survival difference between low, intermediate, and high BACES risk groups, discrimination between individual prognoses was poor (Harrell C-statistic: 0.67, 95% CI 0.57–0.76). However, the calibration curve showed good agreement between predicted and observed all-cause mortality. Modifying the BACES model by adjusting the ESR cut-off and replacing male sex by a history of COPD, improved its discriminatory performance (Harrell C-statistic: 0.76, 95% CI 0.69–0.84).

The original BACES model was developed as a prognostic tool that could be used to guide clinicians during selection of the appropriate treatment strategy for patients with NTM-PD. After its derivation, the model exhibited excellent performance in an internal validation cohort [7]. The poor discriminatory performance observed in our study may be attributable to several factors: i) the relatively small sample size, ii) a higher mortality rate (54% vs. 18%) during a similar follow-up period, iii) the limited predictive value of two BACES components - ESR and male sex (table S2), and iv) several population differences compared to the South-Korean cohort, which resulted in an overrepresentation of patients with intermediate risk and thereby possibly reduced the model's ability to effectively rank-order individuals. For instance, our study population was older (median age 63 vs. 61 years), had a higher proportion of males (44% vs. 36%), and exhibited more severe NTM-PD (cavitary disease in 50% vs. 29%; treatment initiation in 86% vs. 59%). Furthermore, comorbidities in our NTM-PD cohort differ from those in large South-Korean cohorts [5], [6], [12], [13]. For example, a history of tuberculosis infection was less common in our cohort (7% vs. 31–41%), whereas a history of COPD (53% vs. 9%) and cardiovascular disease (31% vs. 7) were more prevalent [14]. In line with COPD studies [15], a history of COPD predicted time to all-cause mortality, and including COPD into the modified BACES model improved its discriminatory performance. Being diagnosed with a cardiovascular disease was excluded from the optimized model due to violation of the proportional hazards' assumption, namely a disproportional (elevated) risk of mortality two years after diagnosis.

In contrast to the poor discriminatory performance, the original BACES model was well-calibrated in our cohort, with only minor improvement in accuracy after modification. While a discrepancy between discrimination and calibration is plausible, the high proportion of patients with active tobacco use, concurrent COPD, cardiovascular disease, or alcohol abuse across BACES risk groups (see Table 1) likely contributed to a substantial baseline mortality risk, which may have diminished the effect of the BACES components on mortality. This could have masked any miscalibration (e.g., underprediction). Both discrimination and calibration must be adequate before a predictive model can be implemented in clinical practice. Consequently, the original BACES model retains utility for group-level risk stratification (e.g., low, intermediate high), but lacks precision for reliably predicting individual-level prognosis in the Dutch cohort.

The external validation study from Canada also demonstrated that the BACES model adequately stratified survival rates among the low, intermediate, and high risk groups. However, contrary to our findings, they reported that the model showed moderate to good discriminatory performance (Harrell C-statistic: 0.73, 95% CI, 0.678–0.787) and miscalibration (i.e., underprediction) of 5-year mortality [8]. Modification of the BMI cut-off slightly improved the model's performance. The authors suggested that population differences, such as ethnicity, age, comorbidity, and the presence of acid-fast bacilli smear positivity at diagnosis, as well as the loss of information due to dichotomization of continues variables, could potentially explain for the suboptimal external performance. The difference in observed mortality rates between our cohort (54%) and the Canadian cohort (18%) along with a more left-skewed BACES score distribution in the Dutch population may contribute to the discrepancy in external calibration performances of the model.

Two studies have explored alternative prediction tools for all-cause mortality in patients with NTM-PD [16], [17]. A Japanese study developed and validated a scoring model similar to BACES, incorporating seven variables: male sex, age ≥ 70 years, BMI <18.5 kg/m2, fibrocavitary disease, malignancy, lymphocytopenia (count <1000 cells/μL), and hypoalbuminemia (<3.5 g/dL) [16]. However, the external performance of this model has not been evaluated. Missing data on lymphocyte count and serum albumin levels, along with a low proportion of patients with active malignancy, currently preclude us from assessing its external performance. Another study from South Korea demonstrated that combining a deep learning model using chest radiography with clinical data (age, sex, BMI, and NTM species) enabled accurate prediction of all-cause mortality [17]. While its applicability in other institutes may be challenging, the use of deep learning to predict prognosis is particularly promising, given the wide spectrum of disease manifestations.

Although culture conversion and microbiological cure rates in our cohort were slightly higher than in previous studies [18], [19], [20], the 5-year mortality rate of 33% falls within the range reported in current literature (10–48%) [20], [21], [22], [23], [24], [25]. Data on recurrence were often missing because most patients were discharged from our clinic after treatment completion.

Despite missing data prohibiting the inclusion of many characteristics in the modified model, univariate cox regression identified several significant predictors (e.g., leukocytosis, and hypoalbuminemia) as well as protectors (e.g., preserved pulmonary function) for time to all-cause mortality (table S1 & S4). These findings are consistent with prior NTM-PD studies [24], [25], [26], [27], and should be considered in future efforts to optimize the model.

This study has two key limitations. First, missing data were common due to the retrospective, single-center design, the study-specific definition for date of NTM-PD diagnosis, as well as the pre-defined time-window during which BACES components were collected. As a result, the performance of the original BACES model could be evaluated in 86 out of 183 (47%) participants, which is substantially lower than in the South-Korean and Canadian cohorts, potentially limiting the statistical power. However, the higher mortality rate in our cohort, may have counterbalanced this limitation. Moreover, the adjusted hazard ratios for significant predictors of time to all-cause mortality (i.e., age, BMI and cavitation) were fairly similar to those from the South Korean cohort, whereas hazard ratios for the non-predictive BACES components (ESR, sex, and acid-fast bacilli smear) were notably lower, suggesting that the observed differences are likely due to true population differences rather than lack of statistical power. Second, duration of follow-up at Radboudumc was heterogeneous due to our role as a reference clinic. Duration and location of follow-up may have affected long-term outcomes.

In conclusion, the original BACES model successfully stratified risk of mortality across its risk groups and was well-calibrated in our NTM-PD cohort from The Netherlands. However, it demonstrated poor discrimination between individual prognoses, possibly due to the relatively limited sample size and population differences between the present and South Korean cohort. Modification of the BACES model, i.e., elevating the ESR cut-off and replacing sex with a history of COPD, improved its discriminatory performance, but further optimization is required. While the original model may be used in The Netherlands for stratifying mortality risk among BACES risk groups, it should not be used for predicting mortality risk in individual patients.

## CRediT authorship contribution statement

**Arthur Lemson:** Writing – original draft, Investigation, Formal analysis, Data curation, Conceptualization. **Esmee van de Pol:** Writing – review & editing, Investigation, Formal analysis, Conceptualization. **Jakko van Ingen:** Writing – review & editing, Supervision, Conceptualization. **Wouter Hoefsloot:** Writing – review & editing, Supervision, Investigation, Conceptualization.

## Ethical approval

The study was exempt from approval by the institutional review board (METC Oost-Nederland; file number: 2024-17038).

## Funding

This research did not receive any specific grant from funding agencies in the public, commercial, or not-for-profit sectors.

## Declaration of competing interest

The authors declare the following financial interests/personal relationships which may be considered as potential competing interests: Jakko van Ingen reports a relationship with Insmed Incorporated that includes: consulting or advisory and travel reimbursement. Jakko van Ingen reports a relationship with An2 Therapeutics Inc that includes: consulting or advisory and travel reimbursement. Jakko van Ingen reports a relationship with Paratek Pharmaceuticals Inc that includes: consulting or advisory and travel reimbursement. If there are other authors, they declare that they have no known competing financial interests or personal relationships that could have appeared to influence the work reported in this paper.
