# Supplementary material for: Performance of the BACES model in a Dutch cohort with nontuberculous mycobacterial pulmonary disease
Source: J Clin Tuberc Other Mycobact Dis. 2026 Jun 27;44:100628. doi: 10.1016/j.jctube.2026.100628 (PMC13377126; doi:10.1016/j.jctube.2026.100628)
Supplement: Supplementary file 1 — Supplementary material [file mmc1.docx]

Supplementary material

Overview of content:

Results:

Figure S1. Distribution of complete BACES scores in the Dutch NTM-PD cohort

Table S1: Additional population characteristics at NTM-PD diagnosis

Table S2: Cox proportional hazard regression of the original BACES model for all-cause mortality

Table S3: Cox proportional hazard regression of the modified BACES model for all-cause mortality

Table S4: Cox proportional hazard regression of other baseline characteristics for all-cause mortality

Figure S1. Distribution of complete BACES scores in the Dutch NTM-PD cohort


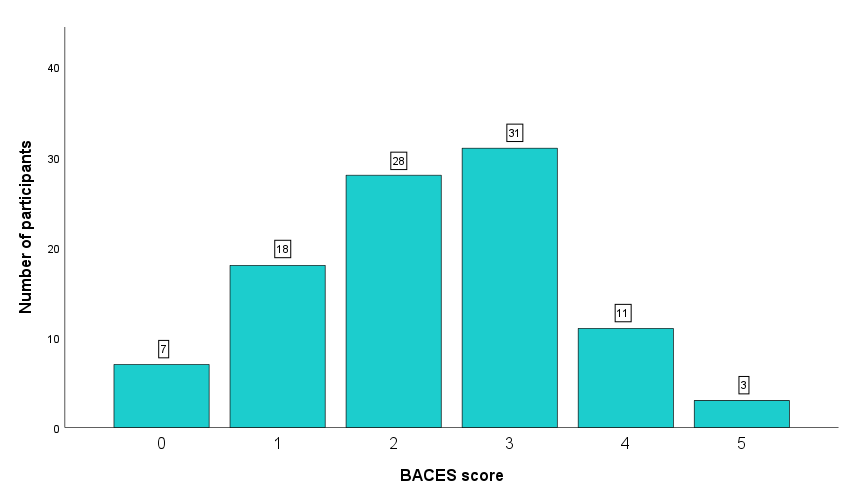


Table S1: Additional population characteristics at NTM-PD diagnosis

| **Characteristic** | **Overall** (N = 183) | **Low risk** (N=28) | **Intermediate risk** (N=75) | **High risk** (N=15) |
| --- | --- | --- | --- | --- |
| **Biochemistry** | | | | |
| CRP mg/l | 13 (4,3-46,8) | 4.5 (1-27.8) | 16 (5-51.5) | 46 (9-93) |
| Hemoglobin, mmol/l | 8,2 (7,6-8,9) | 8.1 (7.7-8.8) | 8.2 (7.7-8.9) | 7.9 (7.2-8.7) |
| Thrombocytes, x10^9^/l | 300 (244,5-397,5) | 306 (244-356.5) | 302.5 (248.3-404) | 425 (273.8-594.8) |
| Leukocytes, x10^9^/l | 8,2 (6,8-10,8) | 6.8 (6-9.3) | 8.9 (7.1-11.3) | 10.2 (6.8-13.3) |
| Absolute lymphocyte count, x10^9^/l | 1,7 (1,3-2,1) | 1.9 (1.3-2) | 1.6 (1,2-2) | 1.5 (1.1-2) |
| Albumin, g/l | 36,0 (32-40,2) | 40 (38-43) | 35 (32-39.6) | 31.5 (27.3-33.8) |
| Aspergillus IgG | 36,5 (23-64) | 44 (29.5-68.5) | 37.5 (24.5-61.8) | 18 (17-33) |
| **Lung function tests** | | | | |
| FEV1, l/sec | 1,7 (1,2-2,4) | 1.7 (1.1-2.4) | 1.6 (1.1-2.3) | 1,8 (1.3-2.7) |
| FEV1% | 61,7 (40,2-82,3) | 66 (44.9-84.5) | 61.3 (40-82) | 53.9 (36.8-79.6) |
| Tiffeneaux Index, FEV/FVC | 56,3 (37-71) | 57.9 (37-68) | 59.6 (41.8-72.8) | 54.5 (39.4-65) |
| DLCO mmol/(min*kPA) | 4,6 (3,2-6,0) | 4.5 (2.8-6.0) | 4.1 (3.2-5.5) | 5.7 (3.1-6.7) |
| DLCO% | 51,9 (39,5-68) | 53.4 (44-77.3) | 50 (38.5-67) | 52.7 (29-70.5) |

*Abbreviations: CRP = C-reactive protein; IgG = Immunoglobulin G; FEV1 = Forced Expiratory Volume in 1 second; FVC = Forced Vital Capacity; DLCO = Diffusing Capacity of the Lungs for Carbon Monoxide*

Table S2: Cox proportional hazard regression of the original BACES model for all-cause mortality

| **Covariate**  (N = 86) | **Unadjusted hazard ratio, 95% CI** | ***p-value*** | **Adjusted regression coefficient** | **Adjusted hazard ratio, 95% CI** | ***p-value*** |
| --- | --- | --- | --- | --- | --- |
| BMI^*^ | 2.19 (1.13-4.24) | 0.02 | 1.09 | 2.98 (1.42-6.25) | 0.00 |
| Age^**^ | 2.79 (1.46-5.32) | 0.00 | 1.38 | 3.99 (1.93-8.22) | 0.00 |
| Cavitary lesion | 1.58 (0.86-2.92) | 0.14 | 0.72 | 2.05 (1.05-4.0) | 0.04 |
| ESR^***^ | 1.62 (0.81-3.21) | 0.17 | 0.24 | 1.27 (0.63-2.56) | 0.50 |
| Male sex | 1.14 (0.62-2.08) | 0.68 | 0.044 | 1.05 (0.55-1.99) | 0.89 |
| AFB positive smear | 0.87 (0.48-1.59) | 0.65 | -0.05 | 0.95 (0.48-1.86) | 0.88 |

^*^ cut-off: BMI < 18.5 kg/m^2^; ^**^ cut-off: age ≥ 65 years; ^***^ cut-off: ESR ≥ 15 mm/h in males and ≥ 20 mm/h in females

Table S3: Cox proportional hazard regression of the modified BACES model for all-cause mortality

| **Covariate**  (N = 86) | **Unadjusted hazard ratio, 95% CI** | ***p-value*** | **Adjusted regression coefficient** | **Adjusted hazard ratio, 95% CI** | ***p-value*** |
| --- | --- | --- | --- | --- | --- |
| BMI^*^ | 2.19 (1.13-4.24) | 0.02 | 1.19 | 3.28 (1.60-6.71) | 0.00 |
| Age^**^ | 2.79 (1.46-5.32) | 0.00 | 1.22 | 3.39 (1.69-6.81) | 0.00 |
| Cavitary lesion | 1.58 (0.86-2.92) | 0.14 | 0.62 | 1.85 (0.97-3.52) | 0.06 |
| ESR^***^ | 2.14 (1.18-3.91) | 0.01 | 0.81 | 2.25 (1.22-4.14) | 0.01 |
| COPD | 2.42 (1.27-4.60) | 0.01 | 0.88 | 2.41 (1.22-4.77) | 0.01 |
| CVD | 2.09 (1.13-3.87) | 0.02 | - | - | - |

^*^ cut-off: BMI < 18.5 kg/m^2^; ^**^ cut-off: age ≥ 65 years; ^***^ cut-off: ESR ≥ 30 mm/h in males and ≥ 45 mm/h in females

Table S4: Cox proportional hazard regression of other baseline characteristics for all-cause mortality

| **Covariate**  (N = 86) | **Unadjusted hazard ratio, 95%CI** | ***p-value*** |
| --- | --- | --- |
| **Biochemistry** |  |  |
| CRP mg/l | 1.00 (1.00-1.01) | 0.37 |
| Hemoglobin, mmol/l | 0.99 (0.82-1.20) | 0.92 |
| Thrombocytes, x10^9^/l | 1.00 (1.00-1.00) | 0.05 |
| Leukocytes, x10^9^/l | 1.14 (1.07-1.22) | < 0.01 |
| Lymphocytes absolute | 1.00 (1.00-1.01) | 0.36 |
| Albumin, g/l | 0.89 (0.84-0.95) | < 0.01 |
| *Aspergillus* IgG | 1.00 (1.00-1.01) | 0.46 |
| **Pulmonary function tests** |  |  |
| FEV1, l/sec | 0.51 (0.34-0.74) | < 0.01 |
| FEV1% | 0.98 (0.97-0.99) | < 0.01 |
| Tiffeneaux Index, FEV/FVC | 0.98 (0.96-0.99) | 0.01 |
| DLCO mmol/(min*kPA) | 0.76 (0.63-0.91) | 0.00 |
| DLCO% | 0.96 (0.94-0.98) | < 0.01 |

*Abbreviations: CRP = C-reactive protein; IgG = Immunoglobulin G; FEV1 = Forced Expiratory Volume in 1 second; FVC = Forced Vital Capacity; DLCO = Diffusing Capacity of the Lungs for Carbon Monoxide*
